# Increasing Trends in Matriculant Publications in the Residency Match: A Scoping Review

**DOI:** 10.7759/cureus.72569

**Published:** 2024-10-28

**Authors:** Henrik A Hahamyan, Nikhil Vasireddi, Bracken Burns, Stephanie A Bousquet, Heath P Gould

**Affiliations:** 1 College of Medicine, Quillen College of Medicine, East Tennessee State University, Johnson City, USA; 2 School of Medicine, Case Western Reserve University School of Medicine, Cleveland, USA; 3 Surgery, Quillen College of Medicine, East Tennessee State University, Johnson City, USA; 4 Obstetrics and Gynecology, Rochester Regional Health, Rochester, USA; 5 Orthopedic Surgery, Hospital for Special Surgery, New York City, USA

**Keywords:** bibliometric analyses, electronic residency applications, medical education, medical student research, residency match

## Abstract

The National Resident Matching Program (NRMP), which is responsible for matching medical students with residency programs in the United States, quantifies an applicant’s research by aggregating their total number of publications, presentations, and abstracts (PPA). However, the program does not differentiate between peer-reviewed publications, which are typically academic studies evaluated by peers in the field, and other types of research output. While several studies have examined the peer-reviewed publications of matriculants in specific specialties, none have compared these specialties to identify trends across the residency match. Comparing peer-reviewed publications across specialties helps the NRMP, medical schools, and applicants identify evolving research expectations and align efforts with specialty-specific benchmarks. Therefore, this scoping review aimed to comprehensively synthesize studies that investigated the peer-reviewed publications of matched medical students. A systematic literature search was performed in September 2023 to identify and extract bibliometric variables from studies analyzing the peer-reviewed publications of matriculated medical students. Of 164 articles screened, 18 studies across 10 specialties were included. Neurosurgery matriculants had the most publications (4.67), whereas ophthalmology had the least (1.23). The proportion of students with zero peer-reviewed publications at application ranged from 22% (neurosurgery) to 47% (orthopedic surgery) and decreased over time for orthopedic and plastic surgery. Publications increased over time for nearly all reported specialties. Higher publication quantity and author H-index were associated with matching into higher-tiered residency programs across all analyzed specialties. The quantity and quality of medical student peer-reviewed publications continue to increase, and higher quality and quantity are associated with matching into higher-tiered programs. Given these trends, medical schools/advisors should continue fostering research mentorship, and students should prioritize both research quantity and quality to optimally prepare for the match. Simultaneously, residency selection committees and policymakers should critically assess whether strong research backgrounds are an optimal method to stratify future physicians and whether there are other avenues to prevent a growing research arms race.

## Introduction and background

Research experience is an increasingly important factor for applicant success in the National Resident Matching Program (NRMP) [1]. The NRMP, which is responsible for matching medical students with residency programs in the United States, quantifies medical student research output by aggregating the number of self-reported peer-reviewed publications, presentations, and abstracts (PPA) [2]. In 2024, U.S. MD- and DO-matched applicants had higher mean PPA than unmatched applicants across nearly all specialties, ranging from 4.2 and 2.9 in family medicine to 37.4 and 23.0 in neurosurgery, respectively [2]. However, peer-reviewed PPA may vary widely in impact, validity, and scientific rigor [3]. Thus, although the PPA offers a standardized metric to quantify an applicant’s general research output, it provides little insight into the quantity and quality of each type of research experience.

Several studies have conducted bibliometric analyses to examine the volume, impact, and patterns of peer-reviewed publications by medical students matching into U.S. residency programs. The primary goal of these studies was to achieve a better understanding of the research profile of applicants who successfully matched into a given specialty by quantitively analyzing the number and quality (i.e. H-index) of each matriculant's publications. To date, researchers have analyzed research output and quality of applicants within specific specialties, but no study has compared across multiple specialties [4,5]. This type of comprehensive synthesis may provide a valuable comparison of research characteristics between specialties while highlighting the overarching trends pertaining to the academic productivity of successful residency matriculants. 

Thus, this review aims to compare the quantity, quality, and characteristics of matriculant peer publications associated with a successful residency match across specialties.

This article was previously presented as a meeting abstract at the 2024 East Tennessee Orthopedics Symposium and 2024 Medical Student Orthopaedic Society on April 6th and 21st, 2024, respectively.

## Review

Material and methods

This scoping review was performed according to Preferred Reporting Items for Systematic Reviews and Meta-Analyses extension for Scoping Reviews (PRISMA-ScR) guidelines [6]. Seven databases (PubMed, CINAHL, SportDiscus, PsycInfo, and Cochrane Databases of Systematic Reviews, Register of Controlled Trials, and Methodology Register) were searched from inception to September 15th, 2023. Research librarians designed search queries with a complex combination of the terms “Medical Students,” “Publications,” “Research,” and “Bibliometrics” to capture studies performing bibliometric analyses among matched medical students. A complete list of search terms per database can be found in the Appendix (Table 5). Duplicates were removed using Rayyan (Rayyan Systems Inc., Cambridge, Massachusetts, USA) [7].

Study Eligibility

Included articles were peer-reviewed, original research that investigated the peer-reviewed publications of residency matriculants for any specialty. Reviews, editorials, letters to the editors, conference abstracts, and non-English studies were excluded. Articles pertaining to research productivity associated with the fellowship match were also excluded, as were articles that reported publications limited to a particular subset of journals or restricted students by residency program ranking. Finally, since the NRMP already reports the aggregate PPA per specialty, articles that grouped peer-reviewed publications with abstracts or presentations in their analysis were excluded [2]. 

Review Process and Data Extraction

An abstract/title review with subsequent full-text review was conducted independently by two reviewers (HAH, NV), with discrepancies being documented and resolved by group consensus in consultation with the senior author (HPG). Reference lists of the included articles were also reviewed to identify any additional articles that satisfied the study eligibility criteria. 

The following characteristics were then extracted from each included article: specialty, matriculating match year, sample size, publication bibliometric characteristics, the relationship of publications to the ranking of matched program, and the number of years from first publication to residency application submission. Matriculating match year was defined as the year that a student began residency. The highest journal impact factor was defined as the single highest journal impact factor in which a matriculant published. The mean journal impact factor was defined as the mean of all journal impact factors in which a matriculant published. 

Statistical Analysis

Although all studies produced quantitative variables with homogenous populations, included papers had notably variable outcomes, methods of data collection, and inclusion/exclusion criteria preventing cumulative meta-analysis. Outcomes were pooled where possible. Any calculations were performed in Microsoft Excel version 16.77.1 (Microsoft, Redmond, WA, USA). 

Results

**Figure 1:**
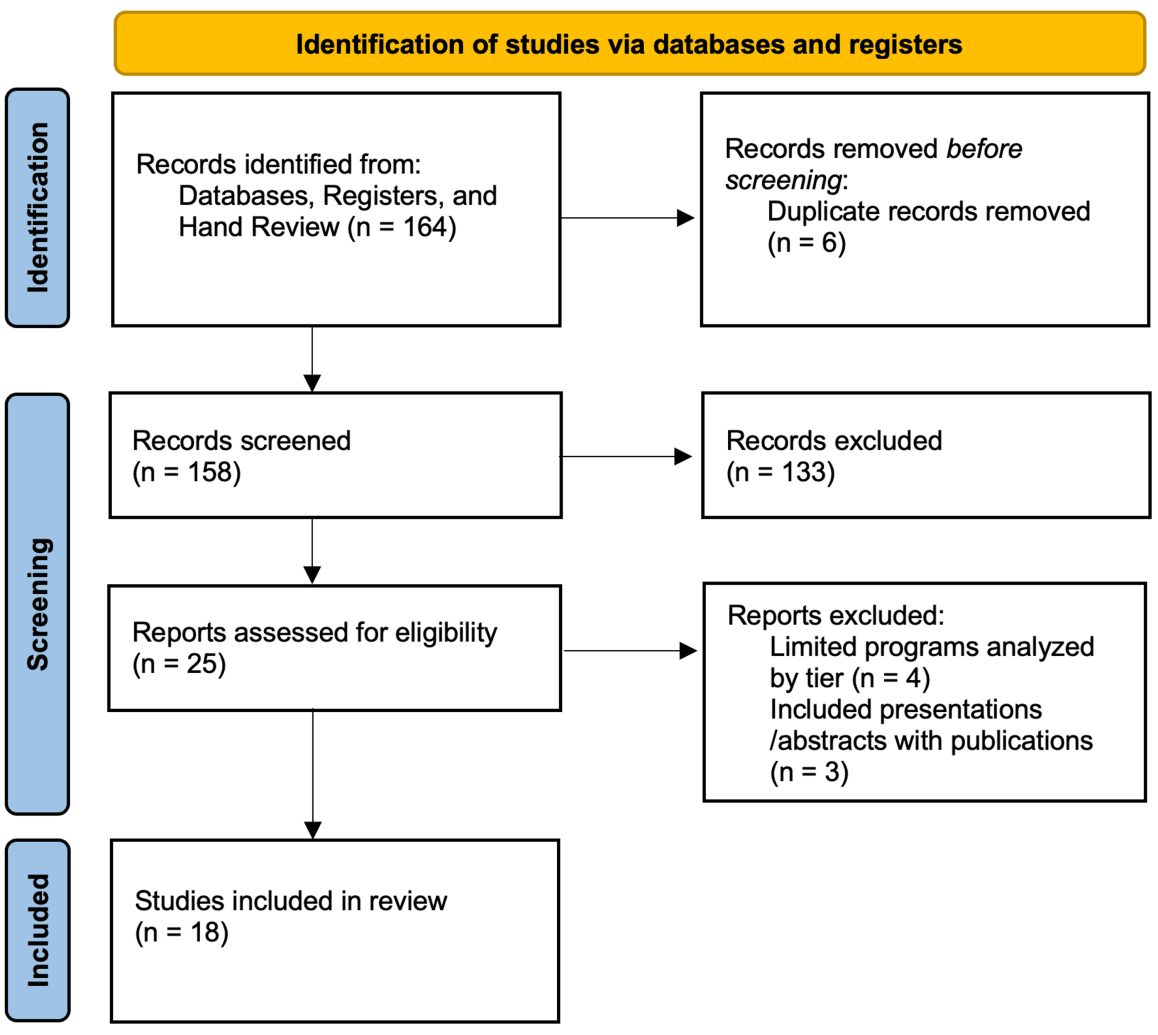
PRISMA diagram PRISMA, Preferred Reporting Items for Systematic Reviews and Meta-Analyses

A total of 18 articles were included in this review (Figure 1), reporting on 10 specialties: dermatology (4), integrated plastic surgery (3), orthopedic surgery (3), neurosurgery (2), otolaryngology (1), integrated vascular surgery (1), integrated interventional radiology (1), ophthalmology (1), radiation oncology (1), and urology (1) [4,5,8-23]. No articles reported on more than one specialty.

Bibliometric Characteristics

**Figure 2:**
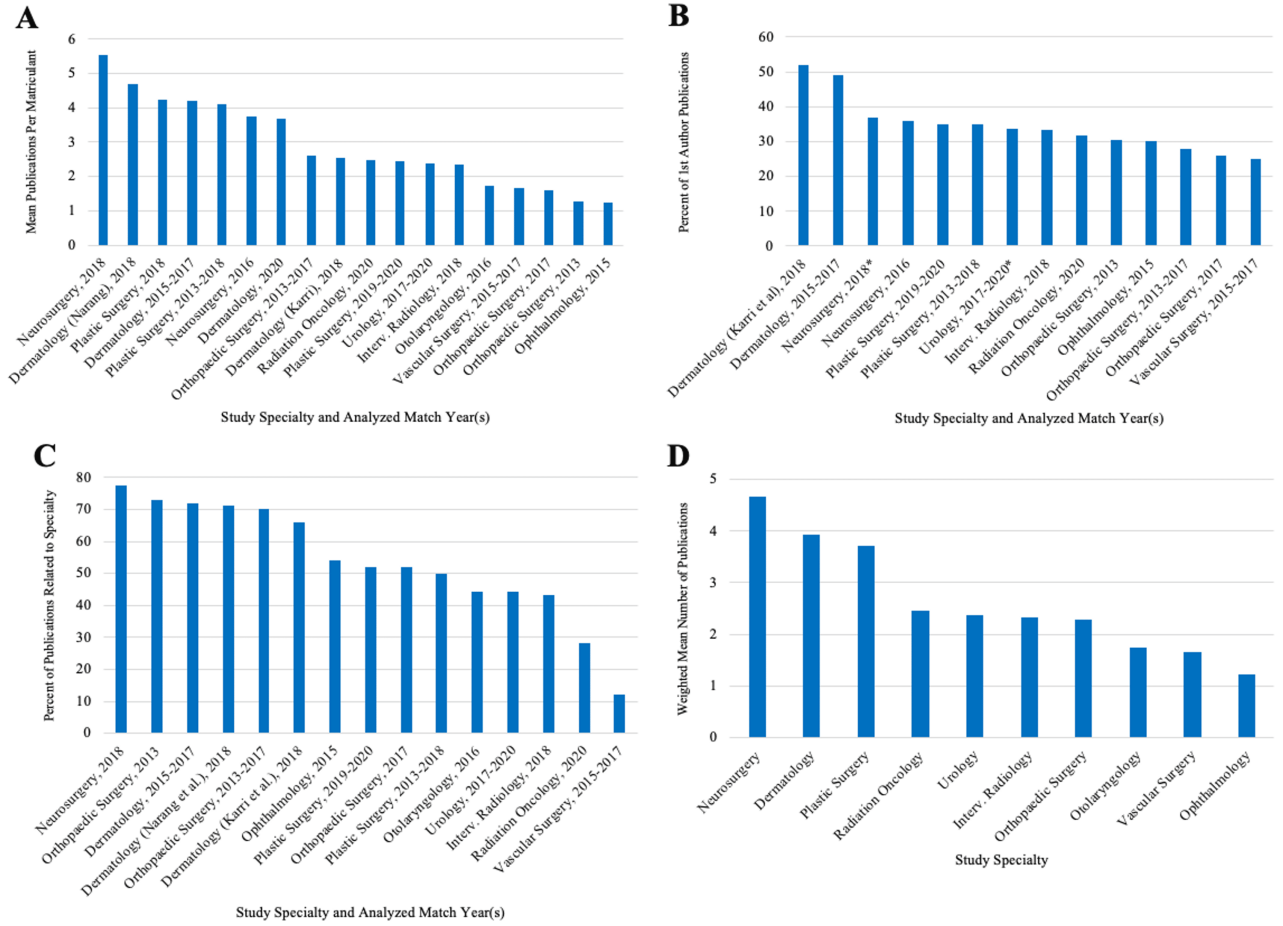
(A) Average number of publications, (B) percent of first author publications, and (C) percent of publications related to specialty across all respective specialties and match years per student. (D) Combined mean number of publications across specialties per student. *Value represents the percentage of combined first and last authorship.

General bibliometric characteristics for all articles are demonstrated (Figure 2, Table 1). Eight studies reported the median number of publications. Vascular surgery, orthopedic surgery (2017, 2013-2017), and urology had a median of 1, whereas neurosurgery (2016) had a median of 1.5, and dermatology (2015-2017) and plastic surgery (2018, 2013-2018) each had a median of two publications per student [4,5,9,10,12,16,18,23]. Nine studies reported temporal trends (Table 2) [8-10,15-19,22,23]. Only urology and integrated vascular surgery reported no significant changes in the mean number of publications over the years analyzed.

**Table 1:** Raw bibliometric characteristics ^a^Other non-sequential years were reported, but 2018 is only listed here. ^b^Value represents the percentage of combined first and last authorship. NR, not reported; SD, standard deviation

Study Author and Year	Specialty	Match Year(s) Investigated	Sample Size, N	Publications, Mean (SD)	1st Author Publications, Mean (%)	Publications Related to Specialty, Mean (%)
Ngaage et al. 2020 [23]	Plastic Surgery	2013-2018	829	4.1 (6.3)	1.44 (35)	2.05 (50)
Oleck et al. 2020 [12]	Plastic Surgery	2018	133	4.25 (7.19)	NR	NR
Mellia et al. 2021 [8]	Plastic Surgery	2019-2020	301	2.43 (3.84)	0.85 (35)	1.31 (52)
Ngaage et al. 2020 [18]	Dermatology	2015-2017	1152	4.2 (NR)	2.06 (49)	3.02 (72)
Narang et al. 2021 [19]	Dermatology	2018^a^	379	4.69 (NR)	NR	3.33 (71.1)
Huang et al. 2022 [13]	Dermatology	2020	401	3.69 (3.7)	NR	NR
Karri et al. 2021 [22]	Dermatology	2018^a^	371	2.55 (0.35)	1.33 (52)	1.68 (66)
Ngaage et al. 2021 [16]	Orthopedic Surgery	2013-2017	3199	2.6 (6.6)	0.73 (28)	1.82 (70)
Campbell et al. 2016 [21]	Orthopedic Surgery	2013	566	1.28 (0.15)	0.39 (30.4)	0.93 (73)
Toci et al. 2020 [4]	Orthopedic Surgery	2017	565	1.6 (3.1)	0.42 (26)	0.83 (52)
Kashkoush et al. 2017 [5]	Neurosurgery	2016	206	3.76 (NR)	1.35 (36)	2.93 (78)
Wadhwa et al. 2020 [15]	Neurosurgery	2018^a^	216	5.54 (8.82)	2.05 (37)^b^	4.30 (77.6)
Thangamathesvaran et al. 2018 [17]	Otolaryngology	2016	222	1.74 (4.32)	NR	0.77 (44.3)
Bargoud et al. 2018 [11]	Ophthalmology	2015	340	1.23 (0.21)	0.37 (30)	0.66 (54)
Bigelow et al. 2021 [9]	Vascular Surgery	2015-2017	158	1.66 (2.39)	0.42 (25)	0.20 (12)
Warren et al. 2020 [10]	Urology	2017-2020	574	2.38 (4.19)	0.80 (33.6)^b^	1.05 (44.1)
Chandra et al. 2019 [14]	Interv. Radiology	2018	117	2.34 (0.41)	0.78 (33.2)	1.01 (43.1)
Huang et al. 2022 [20]	Radiation Oncology	2020	187	2.47 (2.88)	0.78 (31.6)	0.70 (28.3)

**Table 2:** Temporal analysis of bibliometric characteristics ^a^Years were specifically the following: 2007, 2009, 2011, 2014, 2016, 2018. NR, not reported

Study	Specialty	Match Year	Significant Changes in Variables Over Analyzed Match Years				
			Total Publications	First Authorships	Articles Related to Specialty	H-Index	Students With No Publications
Wadhwa et al. 2020 [15]	Neurosurgery	2007-2018^a^	Increase	Increase	NR	NR	NR
Narang et al. 2021 [19]	Dermatology	2007-2018^a^	Increase	NR	Increase	NR	NR
Karri et al. 2021 [22]	Dermatology	2007-2018^a^	Increase	NR	NR	NR	NR
Ngaage et al. 2021 [16]	Orthopedic Surgery	2013-2017	Increase	Decrease	No change	Increase	Decrease
Ngaage et al. 2020 [23]	Plastic Surgery	2013-2018	Increase	No change	No change	Increase	Decrease
Thangamathesvaran et al. 2018 [17]	Otolaryngology	2014-2016	Increase	Increase	Increase	No change	NR
Bigelow et al. 2021 [9]	Vascular Surgery	2015-2017	No change	No change	No change	NR	NR
Ngaage et al. 2020 [18]	Dermatology	2015-2017	Increase	No change	No change	Increase	No change
Warren et al. 2020 [10]	Urology	2017-2020	No change	No change	No change	NR	NR
Mellia et al. 2021 [8]	Plastic Surgery	2019-2020	NR	NR	NR	NR	NR

Eleven articles reported the percentage of students with zero publications prior to residency (Table 3) [4,8-10,14-16,18,21-23]. Thirteen articles compared the mean number of publications to the respective PPA value published by the NRMP (Table 2) [8,9,14,15,19-22]. Of the six articles that reported a median H-index, only neurosurgery had a non-zero value of one [4,5,9,18,16,23]. Six articles reported a mean H-index ranging from 0.96 for interventional radiology to 2.44 for ophthalmology [8,11,12,14,17,20]. Eleven studies provided a breakdown of article type (i.e., original research, review, case report, and commentary) per matriculant, and all reported that original research was the most prevalent. Thirteen articles reported factors associated with matching into a higher-tiered residency program (Table 4) [4,5,8-12,14,15,17,20-22].

**Table 3:** Comparison of publications versus publications/presentations/abstracts and percentage of students with no publication ^a^Other non-sequential years were reported, but 2018 is only listed here. ^b^Reported by Careers in the Medicine report from the AAMC. NR, not reported; PPA, publications, presentations, and abstracts; NRMP, National Residency Matching Program; AAMC, Association of American Medical Colleges

Study	Specialty	Match Year (s) Investigated	Publications, Mean (% of PPA)	PPA by NRMP, Mean	Students With No Publications, N (%)
Wadhwa et al. 2020 [15]	Neurosurgery	2018^a^	5.54 (30)	18.3	48 (22.2)
Huang et al. 2022 [20]	Radiation Oncology	2020	2.47 (13)	18.3	NR
Ngaage et al. 2020 [18]	Dermatology	2015-2017	4.2 (29)	14.7	276 (24)
Narang et al. 2021 [19]	Dermatology	2018^a^	4.69 (32)	14.7	NR
Karri et al. 2021 [22]	Dermatology	2018^a^	2.55 (17)	14.7	111 (29.9)
Mellia et al. 2021 [8]	Plastic Surgery	2019-2020	2.43 (17)	14.2	105 (35)
Oleck et al. 2020 [12]	Plastic Surgery	2018	4.25 (30)	14.2	NR
Ngaage et al. 2020 [23]	Plastic Surgery	2013-2018	4.1 (34)	12.2	232 (28)
Ngaage et al. 2021 [16]	Orthopedic Surgery	2013-2017	2.6 (23)	11.5	1311 (41)
Chandra et al. 2019 [14]	Interventional Radiology	2018	2.34 (28)	8.4	41 (35)
Bigelow et al. 2021 [9]	Vascular Surgery	2015-2017	1.66 (20)	8.3	69 (44)
Toci et al. 2020 [4]	Orthopedic Surgery	2017	1.6 (20)	8.2	266 (47)
Warren et al. 2020 [10]	Urology	2017-2020	2.38	7.7^b^	223 (38.8)
Campbell et al. 2016 [21]	Orthopedic Surgery	2013	1.28 (19)	6.7	279 (49.3)
Kashkoush et al. 2017 [5]	Neurosurgery	2016	3.76	NR	NR
Thangamathesvaran et al. 2018 [17]	Otolaryngology	2016	1.74	NR	NR
Bargoud et al. 2018 [11]	Ophthalmology	2019	1.23	NR	NR
Huang et al. 2022 [13]	Dermatology	2020	3.69	NR	NR

**Table 4:** Association of total publications and author H-index with matching into a higher-tier residency program X represents that a significant association was present. ^a^Tiering based on a research-related ranking, such as top NIH-funded programs, Doximity research rankings, or relative departmental H-index. ^b^Tiering based on a reputation-related ranking, such as Doximity reputation or US News and World Report. NR, not reported

Study	Specialty	Reputation Ranking^a^		Research Ranking^b^		
		Total Publications	Author H-index		Total Publications	Author H-index
Oleck et al. 2020 [12]	Plastic Surgery	-	X		X	X
Mellia et al. 2021 [8]	Plastic Surgery	X	X		X	X
Karri et al. 2021 [22]	Dermatology	NR	NR		-	NR
Campbell et al. 2016 [21]	Orthopedic Surgery	X	NR		X	NR
Toci et al. 2020 [4]	Orthopedic Surgery	X	X		X	X
Kashkoush et al. 2017 [5]	Neurosurgery	NR	NR		X	X
Wadhwa et al. 2020 [15]	Neurosurgery	X	NR		NR	NR
Thangamathesvaran et al. 2018 [17]	Otolaryngology	NR	NR		X	X
Bargoud et al. 2018 [11]	Ophthalmology	X	X		X	X
Chandra et al. 2019 [14]	Interv. Radiology	NR	NR		-	X
Warren et al. 2020 [10]	Urology	X	NR		NR	NR
Bigelow et al. 2021 [9]	Vascular Surgery	NR	NR		X	X
Huang et al. 2022 [20]	Radiation Oncology	X	X		X	X

Specialty Specific Characteristics

Dermatology: Four articles investigated the publications of students matching in dermatology with analysis of over 1152 students in 2015-2017, 401 in 2020, 371 in 2018, and 2234 across 2007, 2009, 2011, 2014, 2016, and 2018 [13,18,19,22]. Cumulatively, there were 3.93 publications per student with 1.95 (49.7%) as first author, 2.78 (70.7%) were dermatology-related, 1.79 (45.6%) were original research, and 387 (25.1%) students held no publications at the time of Electronic Residency Application Service (ERAS) application. Narang et al. and Karri et al. identified a significant increase in the mean number of publications per student (1.6 to 4.7, 203%, 0.8 to 2.6, 325%, respectively) between 2007 and 2018, and Ngaage et al. reported that the median number of publications significantly increased between 2015 and 2017. Only Karri et al. investigated relationships with higher tiers with three separate research rankings and identified no significant associations with total, dermatology-related, or first-author publications. 

Neurosurgery: Two articles analyzed the publications of students matching into neurosurgery representing 206 and 216 matriculants for the 2016 and 2018 match year, respectively [5,15]. Cumulatively, there were 4.67 publications per student with 1.70 (36.5%) as first author and 3.63 (77.8%) neurosurgery/neuroscience-related articles. One study reported that 48 (22.2%) students had no publications at the time of ERAS application for the 2018 match year [15]. For neurosurgery, Wadhwa et al. ranked programs by three separate research-based rankings, and identified that matriculants in the top 40 programs had significantly higher total, neurosurgery-specific, first author, neurosurgery first author, basic science, and clinical publications across all ranking systems [15]. Kashkoush et al. tiered programs (tier 1-5) based on departmental H-index and found that H-index was significantly greater among tier 1 matriculants [5]. 

Orthopedic surgery: Three articles investigated the publications of orthopedic surgery matriculants with a cumulative of 4330 students across the 2013/2017 match years [4,16,21]. The combined mean was 2.30 publications per student, while 0.61 (26.7%) were first-authored, 1.56 (68%) were orthopedic surgery-related, 1853 (42.8%) students had zero publications at the time of ERAS application, and median H-index was 0. Between 2013 and 2017, Ngaage et al. identified that the H-index, total publications, and proportion of clinical research significantly increased, while the proportion of first-authored papers, basic science articles, and of students with zero publications significantly decreased [16]. Two studies investigated factors influencing matching into higher-tier residencies (tier 1-5) based on institution academic performance, NIH funding, and US News ranking [4,21,24]. Toci et al. identified that students in tier 1 programs had significantly higher total publications, H-index, and citations than those in tier 2, but no differences between other adjacent tiers [4]. Students with one publication matched into higher-tier programs compared to those with none, with no significant differences between those with one publication and those with two or more. Campbell et al. identified that more total, first/last author, and orthopedic surgery-related publications were associated with higher-tier programs [21]. 

Plastic surgery - integrated: Three articles included students matching into plastic surgery over the match years 2019-2020, 2013-2018, and 2018 [8,12,23]. Across a cumulative 1263 students, the mean number of publications per student was 3.718, while two studies found a median of two publications per student [12,23]. On average, 2079 (50%) papers were plastic surgery-related and 1443 (35%) were first-authored [8,23]. Two studies reported an average H-index of 1.01 and 1.25, respectively, while the third reported a median H-index of 0 [8,12,23]. Two articles investigated bibliometric characteristics associated with matching into higher-tier programs [8,12]. Oleck et al. tiered programs using top-50 vs. not-top-50 National Institute of Health (NIH) funding and identified significantly higher publications (5.81 vs 2.88, p=0.003) and H-index (1.93 vs. 0.78, p=0.006), respectively. Using both Doximity reputation and research, Mellia et al. identified that H-index, total publications, plastic surgery-related publications, and first-author publications were associated with matching into higher-tiered programs.

Otolaryngology: One article analyzed the publications of students matching into otolaryngology, representing 222 students in 2016 [17]. Thangamathesvaran et al. reported 1.74 mean publications per student where 0.77 (44.3%) papers were otolaryngology-related, 1.48 (85%) were original research, and the mean author H-index was 0.99 with the largest highest mean journal impact factor of 5.04 [17]. When investigating associations with higher-tiered programs defined by Doximity research rankings, author H-index and total number of publications per student were greater in students matching into the highest tier of residency compared to other tiers after multivariate regression [17]. 

Vascular surgery - integrated: One article investigated the publications of 158 students matching into integrated vascular surgery across the 2015-2017 match years [9]. There was a mean of 1.66 and median of one publication per student, with 0.42 (25%) as first author, 1.38 (83%) as original research, a median author H-index of zero, and 65 (41.1%) students with no publications. Vascular surgery had the lowest percentage of papers related to its respective specialty (n=0.20, 12%) [9]. This study also identified that applicants matching into top-10 programs had higher bibliometric characteristics for all variables studied compared to matching into a non-top-10 program when ranked by Doximity research rankings [9]. 

Interventional radiology - integrated: One article included the publications of students matching into integrated interventional radiology, representing 117 students in the 2018 match [14]. There was a mean 2.34 of publications per student with 0.75 (32.2%) as first author, 1.00 (43.1%) as radiology-related, and 41 (35%) students with zero publications prior to beginning residency. Interventional radiology had the lowest mean H-index (0.96), lowest mean highest journal impact factor (2.76), and lowest total mean journal impact factor (1.82) among the five papers that reported these statistics. When investigating bibliometric associations with residency rankings, the number of radiology-related publications, author H-index, and highest journal impact factor were associated with matching into a higher-tiered program when ranked by residency program research output [14]. 

Ophthalmology: One article included the publications of students matching into ophthalmology, which captured 340 matriculants in the 2015 match year [11]. Matriculants had a mean of 1.23 publications, 0.37 (30%) papers were first-authored, 0.66 (54%) were ophthalmology-related, 0.95 (77%) were original research, and 0.11 (9%) were reviews. Although ophthalmology had the lowest mean number of publications per student among specialties, it had the largest mean author H-index among the five papers that reported that statistic. When ranking by institution research output, total number of publications, highest and average journal impact factor, and total H-index were associated with matching into a higher-ranked program. When ranking by program reputation (U.S. News and World Report Ophthalmology Rankings), increased time from first publication to ERAS application was associated with matching into a higher-tiered program.

Radiation oncology: One article investigated the peer-reviewed publications of students matching into radiation oncology representing 187 matriculants from 83 residency programs in the 2020 match [20]. They identified a mean of 2.47 total publications per matriculant where 0.70 (28.3%) were radiation oncology-related, 0.78 (31.6%) were first-authored, and there was a mean author H-index of 1.84. The 2.47 total publications corresponded with a PPA of 18.3, which was the largest discrepancy compared to all other specialties. Total number, specialty-specific, number of first-author publications, and author H-index significantly correlated with a higher tier based on Doximity research and reputation rankings. 

Urology: One article analyzed the publications of students matching into urology in the 2017-2020 match years, representing 574 students across 42 programs [10]. There was a mean of 2.38 and a median of one publication per student, 0.80 (33.6%) were first/last author, 1.05 (44.1%) were urology-related, and 223 (38.8%) students had no publications. Linear regression showed that as the program rank, by Doximity reputation, decreased, there were significantly fewer total publications, number of urology-related publications, and number of first/last authored papers for students in those programs. Furthermore, students who matched at a top-10 program had significantly more publications, more urology-related publications, and more first/last author publications than students matching at non-top-10 programs. 

Discussion

To our knowledge, this scoping review is the first study to comprehensively synthesize the literature pertaining to the publications of medical students who successfully matched into residency across multiple specialties. Cumulatively, the mean number of publications per student ranged from 1.23 for ophthalmology to 4.67 for neurosurgery, and the total number of publications increased across analyzed match years for nearly all included specialties. As anticipated, a greater number of total publications and a higher H-index were associated with matching into a higher-tiered residency program by research rankings across nearly all specialties analyzed. Conversely, many successful residency matriculants had zero peer-reviewed publications at the time of residency application submission.

Besides orthopedic surgery, the top three specialties with the most publications per matriculant (neurosurgery, dermatology, plastic surgery) were the most competitive based on the proportion of NRMP applicants that went unmatched in 2023 [25]. The competitiveness of a specialty and quantity of peer-reviewed research are likely correlated because research publications may be interpreted (whether appropriately or inappropriately) as a surrogate for an applicant’s longitudinal interest in the field, academic work ethic, and potential to contribute to a department’s research productivity after matriculation [26,27]. Despite this, findings of relatively high rates of students with zero peer-reviewed publications at ERAS application submission were unexpected given the competitive nature of these specialties and underscore the continued importance of non-publication-based aspects of a medical student’s background (i.e., letters of recommendation, personal statement, clinical performance) in the residency match process [28].

When investigating the known PPA value reported by the NRMP, all articles that compared the mean number of publications to the PPA value identified that the number of peer-reviewed publications was lower, although no formal statistical analysis was performed. Therefore, it appears that this compilation of PPA into a single value emphasizes pure quantity as opposed to credible, peer-reviewed scholarly work. For example, one applicant with 10 impactful, peer-reviewed publications versus another applicant with 10 local/regional conference posters would be viewed as academically equal within the limits of the PPA, while the inherent quality of the work may be substantially different [29]. Although this statistic may still represent a strong research background, this phenomenon illustrates that medical students should not use mean PPA to estimate the number of peer-reviewed publications required to successfully match their desired specialty.

Beyond publication count, H-index also increased over time for orthopedic surgery, plastic surgery, and dermatology. This consistent trend across competitive specialties underscores an “academic arms race” with regard to the importance of publications in the residency match process [15]. Since H-index is a well-established measure of research quantity and quality, this trend is likely a by-product of increased preference for students with more research products [30]. Medical students should prioritize attaining a higher H-index in order to maximize their chances of matching into a higher-tier residency program.

Several limitations to this review are identified. The included articles only represent the data among successfully matched students in their respective specialties, as opposed to matched versus unmatched students, thereby limiting our ability to draw conclusions regarding factors that may be associated with matching successfully. Beyond this, “higher tier” programs are traditionally considered to be those with a strong academic/research focus, but these may not reflect all applicants’ preferences. Advisors and students should contextualize these results to their own situation and what they seek in their training. This review also includes only 10 specialties, with a clear bias toward those with the most competitive match rates. Thus, the quantity and quality of matriculant publications reported here should not be generalized to other specialties and may not represent the entire match [25]. Furthermore, all analyzed articles were published before the conversion of the United States Medical Licensing Exam Step 1 to a pass/fail score system. Given that a pass/fail system reduces a variable that could have been used to differentiate applicants, a larger focus on remaining differentiating characteristics, such as research, may be emphasized in the coming years. Additional bibliometric analyses should be performed to provide timely updates on previously analyzed and to explore new specialties. 

Moving forward, residency selection committees should consider whether these research-heavy criteria are an optimal means of stratifying prospective physicians and surgeons in their specialty. In the interim, as research output continues to play a pivotal role in the residency match, we recommend that the NRMP separate peer-reviewed publications from presentations and abstracts, add measures of quality (e.g., author H-index), and provide avenues for applicants to better convey how their research experiences align with their future academic goals. 

## Conclusions

Given increasing total publications and author H-index over time across nearly all specialties, this scoping review underscores the increasing presence of an “academic arms race” that has become even more prevalent over time. Although peer-review publications remain a small portion of the total number of PPA reported by the NRMP, their higher quantity and quality continue to be associated with higher-tiered residency programs. Medical schools and advisors should continue to support early research mentorship while establishing programs to teach how to perform research accurately, and medical students should begin seeking research early while focusing on both quantity and quality to increase the chances of success in the residency match.

## Appendices

**Table 5:** Systematic search terms

Database	Search Entry
PubMed	(("Schools, Medical"[Mesh] OR "Education, Medical, Graduate"[Mesh] OR Medical[tiab]) AND ("Students"[Mesh] OR Student*[tiab]) OR ("Students, Medical"[Mesh])) AND ("Publishing"[Mesh] OR "Publications"[Mesh] OR "Research"[Mesh] OR research*[tiab] OR Publish*[tiab]) AND ("Bibliometrics"[Mesh] OR Bibliomet*[tiab])
CINAHL	(((MH "Schools, Medical") OR (MH "Education, Medical+") OR ((ti(medical)) OR (ab(medical)))) AND ((MH "Students+") OR ((ti(student*)) OR (ab(student*))) OR (MH "Students, Medical+")) AND ((MH "Publishing+") OR (MH "Research+") OR (ti(publish*)) OR (ab(publish*)) OR (ti(research*)) OR (ab(research*))) AND((MH "Bibliometrics+") OR (ti(bibliomet*)) OR (ab(bibliomet*))))
SportDiscus	(DE "MEDICAL students" OR (ti(medical)) OR (ab(medical))) AND (DE "STUDENTS" OR DE "COLLEGE students" OR DE "MEDICAL students" OR DE "SCHOOL children" OR DE "STUDENTS with disabilities" OR (ti(student*)) OR (ab(student*))) AND ((ti(publish*)) OR (ab(publish*)) OR (ti(research*)) OR (ab(research*)))AND ((ti(bibliomet*)) OR (ab(bibliomet*)))
PsycInfo	(DE "Medical Education" OR DE "Medical Internship" OR DE "Medical Residency" OR DE "Psychiatric Training" OR (ti(medical)) OR (ab(medical))) AND ((DE "Medical Students" OR DE "Students" OR (ti(student*)) OR (ab(student*))) AND ((ti(publish*)) OR (ab(publish*)) OR (ti(research*)) OR (ab(research*))) AND (DE "Bibliometrics" OR (ti(bibliomet*)) OR (ab(bibliomet*)))
Cochrane Database of Systematic Reviews	(((ti(medical)) OR (ab(medical))) AND ((ti(student*)) OR (ab(student*))) AND ((ti(publish*)) OR (ab(publish*)) OR (ti(research*)) OR (ab(research*))) AND((ti(bibliomet*)) OR (ab(bibliomet*))))
Cochrane Central Register of Controlled Trials	(((ti(medical)) OR (ab(medical))) AND ((ti(student*)) OR (ab(student*))) AND ((ti(publish*)) OR (ab(publish*)) OR (ti(research*)) OR (ab(research*))) AND((ti(bibliomet*)) OR (ab(bibliomet*))))
Cochrane Methodology Register	(((ti(medical)) OR (ab(medical))) AND ((ti(student*)) OR (ab(student*))) AND ((ti(publish*)) OR (ab(publish*)) OR (ti(research*)) OR (ab(research*))) AND((ti(bibliomet*)) OR (ab(bibliomet*))))
